# Using decision-analysis modelling to estimate the economic impact of the identification of unrecognised bipolar disorder in primary care: the untapped potential of screening

**DOI:** 10.1186/s40345-022-00261-9

**Published:** 2022-06-10

**Authors:** Jiri Benacek, Nayra A. Martin-Key, Benedetta Spadaro, Jakub Tomasik, Sabine Bahn

**Affiliations:** Department of Chemical Engineering and Biotechnology, University of Cambridge, Philippa Fawcett Drive, Cambridge, CB3 0AS UK

**Keywords:** Misdiagnosis, Bipolar disorder, Screening, Economic impact, Decision analysis modelling

## Abstract

**Background:**

Patients with bipolar disorder are often unrecognised and misdiagnosed with major depressive disorder leading to higher direct costs and pressure on the medical system. Novel screening tools may mitigate the problem. This study was aimed at investigating the direct costs of bipolar disorder misdiagnosis in the general population, evaluating the impact of a novel bipolar disorder screening algorithm, and comparing it to the established Mood Disorder Questionnaire. A decision analysis model was built to quantify the utility of one-time screening for bipolar disorder in primary care adults presenting with a depressive episode. A hypothetical population of interest comprised a healthcare system of one million users, corresponding to 15,000 help-seekers diagnosed with major depressive disorder annually, followed for five years. The model was used to calculate the impact of screening for bipolar disorder, compared to no screening, in terms of accuracy and total direct costs to a third-party payer at varying diagnostic cut-offs. Decision curve analysis was used to evaluate clinical utility.

**Results:**

Compared to no screening, one-time screening for bipolar disorder using the algorithm reduced the number of misdiagnoses from 680 to 260, and overall direct costs from $50,936 to $49,513 per patient, accounting for $21.3 million savings over the five-year period. The algorithm outperformed the Mood Disorder Questionnaire, which yielded 367 misdiagnoses and $18.3 million savings over the same time. Decision curve analysis showed the screening model was beneficial.

**Conclusions:**

Utilisation of bipolar disorder screening strategies could lead to a substantial reduction in human suffering by reducing misdiagnosis, and also lessen the healthcare costs.

**Supplementary Information:**

The online version contains supplementary material available at 10.1186/s40345-022-00261-9.

## Background

### Burden of bipolar disorder

Bipolar disorder (BD) is a common and disabling chronic disease characterised by intermittent manic or hypomanic and depressive episodes. It is often associated with significant impairments that impact personal, social, and occupational functioning, imposing burdens on individuals and healthcare systems alike. It is estimated that in the United States alone, BD affects more than three million individuals (1.8% of the population) in any given year, with an estimated lifetime prevalence of up to 4.1% (Kessler et al. 2012). Ranked among the leading causes of disability worldwide (Whiteford et al. 2015), the disorder’s impact is reflected in its substantial economic burden, which, according to a recent systematic review, could be as high as $195 billion annually in the US alone (Bessonova et al. 2020). Of this, approximately 25% are direct medical costs such as treatment and hospitalisations, and the remaining 72% to 80% are indirect costs such as loss of productivity and unemployment.

A factor that significantly contributes to the costs associated with BD and other psychiatric conditions is the lack of objective diagnostic tests. Diagnostic practice in psychiatry focuses on the identification of overlapping symptom profiles and is dependent on the reliability of psychometric instruments and expert consensus rather than aetiology and pathophysiology. This, as well as the fact that BD frequently presents with comorbidities, can make the correct diagnosis challenging (Singh and Rajput 2006). Most BD patients initially present with a depressive episode (Mitchell et al. 2008), with hypomanic episodes being highly under-reported because periods of high energy may not be experienced negatively by the patient (Singh and Rajput 2006). Additionally, the episodes of depression are usually more frequent and longer-lasting than manic episodes (Tondo et al. 2017; Judd and Akiskal 2003). For these reasons, BD may be initially misdiagnosed as major depressive disorder (MDD), as observed in 37% of cases (Ghaemi et al. 2000). BD patients report on average 5.7–7.5 years delay between the onset of symptoms and an accurate diagnosis (Morselli and Elgie 2003; Ghaemi et al. 1999). During this time, they consult on average four professionals and remain either mis- or undiagnosed (i.e., unrecognised) (Hirschfeld and Vornik 2003).

### Impact of misdiagnosis of bipolar disorder

Misdiagnosis can have severe consequences for the course and severity of BD, and treatment outcomes. This can range from treatment being less effective, to it actively facilitating further deterioration. Untreated or mistreated BD has been found to result in an increase in the number of hospitalisations, suicide attempts and completion rates, the exacerbation of manic episodes, and increased frequency of rapid cycling BD (Altamura et al. 2010). Critically, a strong link between rapid cycling and antidepressant use has been observed, with treated patients being almost four times more likely to experience rapid switching of episodes, associated with medication non-compliance, and increased the number of psychiatric appointments (‘revolving door’ patients) (Schneck et al. 2008). Unfortunately, in primary care settings, where most patients initially seek help, selective serotonin reuptake inhibitors (SSRIs) and other antidepressants are usually the first-line intervention for depressive symptoms (Johnson et al. 2017).

Although there is a consensus that misdiagnosis of BD increases the overall costs of healthcare (Bessonova et al. 2020), in-depth economic evaluations estimating the true degree of its impact are scarce. The treatment pathways in mental healthcare tend to be both overlapping between different diagnoses sharing similar symptoms (McIntyre and Calabrese 2019), and highly variable. Treatment for the same diagnosis depends on a patient’s symptoms, drug response and side-effects and, thus, can vary substantially (Olbert et al. 2014). Additionally, studies examining the economic impact of an illness often employ heterogeneous methodologies and their estimates are thus highly variable due to different target populations and cost categories (Bessonova et al. 2020; Kleine-Budde et al. 2014). Although such studies offer valuable insights into various burdens of misdiagnosis in their respective contexts, their cross-comparability is low and thus inferring costs of misdiagnosis from secondary data is complicated.

### Screening and its potential in mental health

While structured psychiatric interviews and diagnostic manuals such as the Diagnostic and Statistical Manual of Mental Disorders, 5th Edition (DSM-5) (APA 2013) or the International Statistical Classification of Diseases and Related Health Problems, 11th Revision (ICD-11) (WHO 2018) are considered a gold standard within psychiatry, their systematic use in primary care, where most diagnoses of depression are made, is limited (Cabana et al. 1999). Given the high burden resulting from misdiagnosis, an effective way of screening for BD could facilitate earlier effective treatment and prevent both the added costs, as well as a substantial amount of human suffering. Despite their accessibility, adoption of screening methods into clinical practice is slow and tentative, with ‘The National Institute for Health and Care Excellence’ (NICE) directly advising against using questionnaires of any kind for the identification of BD in adults (NICE 2019). However, in a previously published study, a one-off screening of 1000 patients newly presenting with a depressive episode using the Mood Disorder Questionnaire (MDQ) (Hirschfeld 2002) has been argued to not only have the potential to save $1.94 million in direct costs over five years, but also facilitate the correct diagnosis of an additional 8% of patients Menzin et al. (2009).

We recently developed a novel digital diagnostic platform for detecting BD in patients with a recent diagnosis of MDD (Tomasik et al. 2021). Data used for model training and validation were collected as a part of the Delta Study, a project undertaken to improve the diagnosis of mood disorders in individuals presenting with low mood (Olmert et al. 2020). The utilised screening tool was based on Extreme Gradient Boosting (XGBoost), a tree-based machine learning algorithm, chosen for its good performance, combined with high explainability, ability to handle missing values, and its intuitive structure. Compared to previous studies on the subject, Delta Study investigated a larger number of participants, employed a more rigorous validation, and is unique due to its ambition to combine symptom and biomarker data using explainable machine learning for easier interpretation of results by medical professionals. The Delta Study algorithm showed good to excellent performance in distinguishing BD from MDD and low mood groups in the study population. However, the impact of utilising the platform in the general population, in terms of the number of averted misdiagnoses and direct costs savings, remains unknown. To this end, the aim of the present study was threefold: (1) to review the literature concerning the costs associated with BD misdiagnosis, (2) to estimate the impact of using the Delta Study algorithm in primary care based on results from the literature review, and (3) to compare the impact of the Delta Study algorithm to a non-screening scenario and to screening with MDQ.

## Methods

### Cost of BD misdiagnosis

To gain better insight into direct costs of misdiagnosing BD as MDD, we searched previously published studies and systematic reviews on the subject. The population of interest was adults aged 18+ years presenting with a depressive episode in a primary care setting, and the costs were researched from the perspective of a third party payer, e.g. a healthcare system or an insurance company. We performed a search on PubMed and Google Scholar, using search terms ’(burden OR economic burden OR price OR cost OR costs OR economi* OR impact OR consequences) AND (undiagnos* OR unrecognis* OR misdiagnos*) AND (bipolar OR mani*)’. Results were reviewed, and cost estimates as well as additional sources from relevant records’ reference lists were then extracted.

### Screening models

*Delta Study algorithm* The Delta Study machine learning model was developed using information from a self-administered purpose-built online mental health questionnaire based on existing diagnostic manuals (APA 2013; WHO 2018), input from psychiatrists, and a range of health screening questionnaires, including MDQ (Sheehan et al. 1998; Hirschfeld 2002; Ghaemi et al. 2005), altogether amounting to 635 distinct questions. Participants were only asked questions relevant to them, reducing the total amount of questions asked to a maximum of 382, with 284 questions asked on average. Median start-to-finish time to answer in full was 46 minutes, with an option to complete it in multiple sittings to make it more convenient for the responders. The questions were divided into six sections: (1) demographic information; (2) bipolar/manic and hypomanic symptoms; (3) depressive symptoms; (4) personality traits; (5) history of medication, treatment, and substance use; and (6) other psychiatric conditions (Olmert et al. 2020). Additionally, dried blood samples were collected to allow for analysis of biomarkers previously associated with psychiatric conditions. Mood disorder diagnoses were determined using the Composite International Diagnostic Interview (CIDI), version 3.0 (Kessler and Üstün 2004). This data was used to develop a diagnostic machine learning model (Tomasik et al. 2021). The number of predictive features selected by the algorithm was variable, however model performance was primarily dependent on five features present in most models: elevated mood, grandiose delusions, talkativeness, recklessness, and risky behaviour.

The model showed an excellent ability to differentiate misdiagnosed participants with BD from those with MDD, with an average area under the receiver operating characteristic curve (ROC AUC) of 0.92. This corresponded to an overall mean accuracy of 83%, with a sensitivity (i.e., ability to identify BD misdiagnosed as MDD) of 84%, and a specificity (i.e. ability to confirm MDD) of 83%. See Additional file 1: A.1 for the full list of the algorithm’s sensitivities and specificities at varying diagnostic cut-off points (i.e. minimal scores needed to be flagged positive for the screened condition).

*Mood disorder questionnaire* The Mood Disorder Questionnaire (Hirschfeld 2002) is a screening tool for BD designed to be utilized in primary care settings. It comprises 13 questions, as well as items assessing functional impairment and clustering of symptoms. Because it is intended as a screening tool and not a diagnostic instrument, positive result should prompt full clinical evaluation and, ideally, referral of the patient to a specialist for psychiatric assessment.

The MDQ at the recommended threshold shows a very good specificity, but relatively low sensitivity scores, with the primary literature reporting an ability to identify seven out of ten patients with BD, while nine out of ten patients without BD would be correctly screened out (Hirschfeld 2002). In the past, it was argued that a screening tool should achieve at least 90% sensitivity. However, after shifting the cut-off to achieve this result, its positive predictive value drops to a level where its use could not be supported in practice (Zimmerman et al. 2011). For the purposes of the current paper, we refer to MDQ’s performance at different cut-offs determined in a primary care setting by Hughes (2016). See Additional file 1: A.2 for the full list of the MDQ’s sensitivities and specificities at varying diagnostic cut-off points.

### Decision-analysis model

The core framework used for appraisal of the novel digital BD screening tool was modelled in Microsoft Excel, as adapted from a decision analysis model used in a previously published study (Menzin et al. 2009). We aimed to estimate the economic utility of using a digital screening questionnaire for mood disorders in primary care, calculated from the third-party payer perspective in the US. It was assumed that the one-year incidence of major depressive episode is 3% (Ferrari et al. 2013) and that approximately 50% of affected individuals would visit a physician’s office because of these symptoms. Assuming a hypothetical healthcare plan of one million people, the model was thus populated with 15,000 individuals presenting with depressive symptoms representing the relevant target population. The structure of the model remained unchanged compared to the study by (Menzin et al. 2009), however most of the input parameters were updated with most recent evidence. For the more detailed information about the model’s structure, please refer to Additional file 1.

In brief, the model defined three categories of interest, i.e., MDD, recognised bipolar patients (RBP), and unrecognised bipolar patients (UBP), as three distinct states with defined transition probabilities, and subsequently followed their diagnostic transition for a period of 5 years. The annual discount rate was set to 3% (Haacker et al. 2020). Transition probabilities differed between the two case scenarios of (a) one-time screening, and (b) no screening. In the screening scenario, all individuals were screened, and positive instances were assigned to either true positive or false positive BPD branches. The rates for all three states were calculated based on likelihoods of receiving a positive or negative result, and the screening test’s positive and negative predictive values at a given cut-off. Additionally, individuals who screened positive were assumed to have a 75% likelihood of visiting a psychiatrist who would either confirm or contest the diagnosis with 100% certainty (e.g., spot misdiagnosis), serving as a gold standard. If the diagnosis reached a correct state, its transition probability was set to 0%, and it was assumed that the patient would remain in that health state for the duration of the follow-up. If a patient left the healthcare plan, they would stop accumulating costs. Costs for each of the three states were based on the literature review. The incidence rate of UBP newly presenting with a depressive episode was assumed to be 16% (Angst et al. 2011). In both scenarios, there was a yearly 15% chance of switching from an incorrect to a correct diagnosis, corresponding to the median delay of 6 years in BD diagnosis. Additionally, there was an assumed attrition rate (all-cause health plan disenrollment) of 10.6% (Government Accountability Office 2017). Finally, we also accounted for one-time costs related to the administration of the screening questionnaires/digital tool, as well as the psychiatric evaluations, which were calculated to be $15, and $230 per patient, respectively.

The model was then used to explore the performance of the screening algorithms at different diagnostic thresholds to find the optimal diagnostic cut-off in terms of (a) the highest savings, and (b) the highest number of correctly diagnosed patients. A range of sensitivity analyses were then performed to evaluate the influence of each core assumption on the model output, and the algorithm performance was compared to the similarly formulated best performing cut-off for the MDQ.

### Decision curve analysis

To evaluate the Delta Study algorithm’s suitability for clinical decision making at the identified diagnostic threshold ranges, we employed a decision curve analysis (Vickers and Elkin 2006). While traditional statistical measures can be used to describe performance of a diagnostic model, the decision curve analysis provides a simple means for evaluation of its clinical utility using a single net benefit value, combining the number of true positives and false positives into a single ’net’ number. The tool’s net benefit is then compared to the utility of both ’none-positive’ and ’all-positive’ scenarios to assess its added value.

## Results

### Cost of BD misdiagnosis

The searches of the existing literature on the subject identified five previous reports estimating the costs of unrecognised BD (Table 1). All the studies were based on medical claim data and were published between 2002 and 2007, with data collected between 1993 and 2004. There was a high variability in reported findings, with reports showing high relative differences in annual costs of unrecognised BD ranging from $161, to $5044 per patient, because of different methodologies used. This is despite the fact that the majority of the identified publications analysed similarly sourced data. Although it would be desirable to refer to more recently published findings, to our knowledge, these are the only sources of detailed information on the costs of BD misdiagnosis to date.

Of the five studies, we used the costs as determined by Birnbaum et al. (2003) in the following decision analysis modelling, owing to its superior population sample, as well as listing the costs for all three categories as opposed to merely relative costs. This equated to $9612, $7020, and $14,148 for RBP, MDD, and UBP, respectively (Table 1). These values were adjusted for inflation, and expressed as 2021 values. The direct per-patient costs of RBP and MDD used in the study were thus $16,092 and $11,760, respectively. The direct cost of UBP was $23,696 per patient, and in the study, it was assumed that misdiagnosed MDD has the direct costs equal to UBP.

**Table 1:** Studies evaluating the economic impact of misdiagnosing BD as MDD, as represented by mean annual per-patient costs

Added annual cost of misdiagnosis studies							
Study	N	Year of collection	Correctly diagnosed		Misdiagnosed	Relative costs	
			RBP	MDD	UBP	UBP vs. RBP	UBP vs. MDD
Li et al. (2002)*	3349	1994–1999	–	–	–	$5044	–
Shi et al. (2004)*	25,460	1993–1999	–	–	–	$682	$995
Matza et al. (2005)	2307	2000	$8600	$7288	$8761	$161	$1373
Birnbaum et al. (2003)	9009	1998–2001	$9612	$7020	$14,148	$4536	$7128
McCombs et al. (2007)*	14,809	1993–2004	–	–	–	$2316	$1681

Relative costs show the difference in costs between correctly diagnosed patients and UBP. Reported costs reflect values as published in the respective studies and have not been adjusted for inflation. N: Sample size; BD: Bipolar disorder; MDD: Major depressive disorder; RBP: Recognised bipolar disorder patients; UBP: Unrecognised bipolar disorder patients

*Studies using Medi-Cal data

### Economic impact

In the default, no-screening scenario, the total direct costs of healthcare in the analysed population equated to $764.0 million over the five-year period, corresponding to an average cost of $50,936 per patient. After accounting for all-cause attrition, during the five-year period the number of patients dropped from the initial 15,000 to 9582. Of those, 8901 patients were correctly diagnosed and 680 patients remained misdiagnosed after five years.

**Fig. 1:**
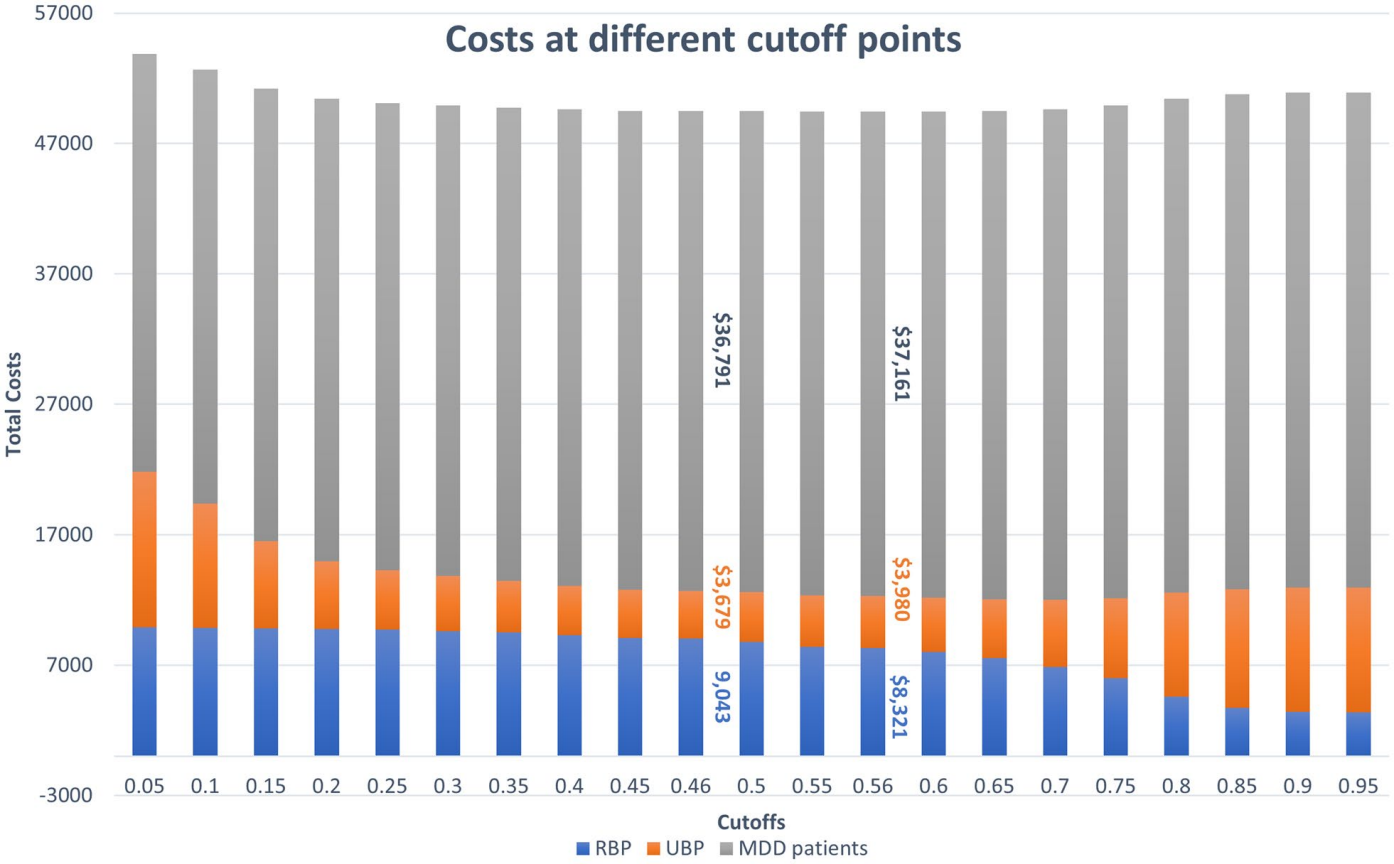
Performance at different cutoffs. Total 5-year inflation-adjusted per-person discounted costs using the Delta Study algorithm (*y* axis) at different cut-off points (*x* axis). Indicated are values for cut-offs at 0.05 intervals as well as the cut-offs with the best performance in terms of number of correct diagnoses (0.46, on the left), and in terms of cost-savings (0.56, right). Grey parts of the stack graph represent costs for correctly diagnosed MDD, orange parts represent costs for UBP/misdiagosis, and blue parts reflect costs for correctly diagnosed BD. BD: Bipolar disorder; MDD: Major depressive disorder; RBP: Recognised bipolar disorder; UBP: Unrecognised bipolar disorder

**Fig. 2:**
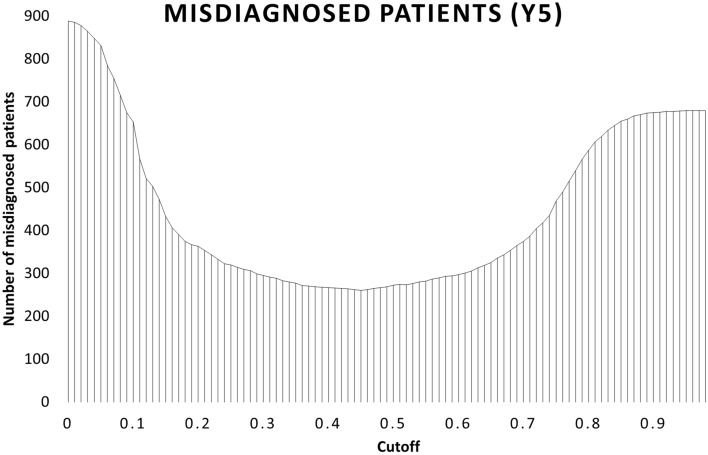
Misdiagnosed at different cutoffs. Total number of misdiagnosed patients at the end of the 5th year of follow-up (*y* axis) at different cut-off points (*x* axis) for the Delta Study algorithm

**Fig. 3:**
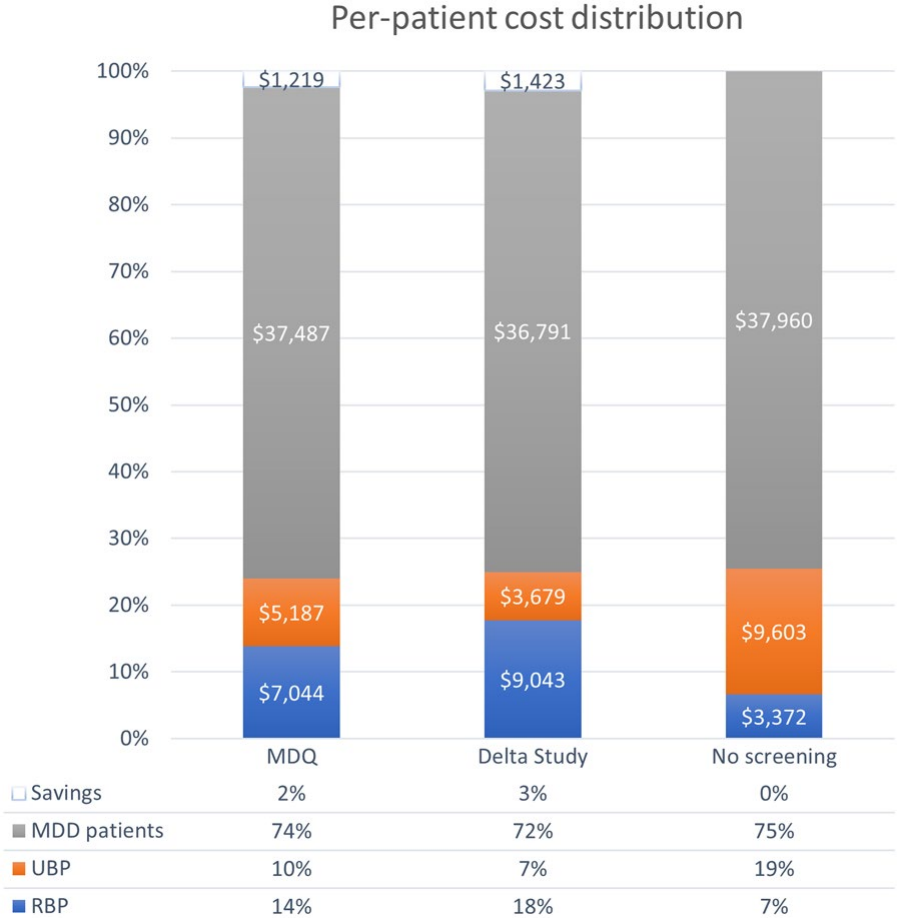
Cost distributions. Total five-year distribution of inflation-adjusted per-person discounted costs. Grey colour represents costs for correctly diagnosed MDD, orange represents costs for UBP, and blue represents costs for RBP. MDD: Major depressive disorder; RBP: Recognised bipolar disorder patients; UBP: Unrecognised bipolar disorder; MDQ: Mood disorder questionnaire

Decision analysis modelling at varying diagnostic cut-offs for the Delta Study algorithm revealed that the lowest direct costs were incurred at the cut-off point of 0.56 (Fig. 1), corresponding to a sensitivity of 0.76 and a specificity of 0.87 (see Fig. S1 in Additional file 1: A1 for the Delta Study algorithm’s performance at different cut-offs.). At this cut-off, the overall direct costs were $741.9 million, or $49,462 per patient, and the number of correct diagnoses and misdiagnoses was 9300 and 282, respectively, after five years (Fig. 2). This represented savings of $22.1 million, or $1474 per patient, over 5 years, and 398 (4.2%) more correct diagnoses compared to the no-screening scenario. The highest number of correctly diagnosed patients, regardless of the diagnosis, was observed at the cut-off point of 0.46, with a sensitivity of 0.87, and a specificity of 0.81. At this cut-off, 9321 patients were correctly diagnosed with MDD or BD at the end of a five-year follow-up period, while 260 patients remained misdiagnosed (Fig. 2). This corresponded to 420 (4.4%) more correct diagnoses relative to no-screening. Total direct costs at the cut-off of 0.46 equated to $742.7 million, or $49,513 per patient over five years (Fig. 1), and represented savings of $21.3 million, or $1423 per person, compared to no-screening (Fig. 3). The total difference in direct costs between the most profitable and most accurate cut-offs for the Delta Study algorithm was $765,000, or $51 per person, over the five-year period. For the following analyses, the threshold of 0.46 was used.

The introduction of one-off screening with the Delta Study algorithm also resulted in the redistribution of overall costs among the three disease states, i.e., MDD, RBP, and UBP. Compared to no-screening, there was a decrease of 3% of the total per-person costs attributed to MDD patients, because of the higher number of false positives. Conversely, the algorithm’s higher sensitivity allows it to save an additional 12% of total per-person costs by reducing the number of UBP, decreasing the sum spent on misdiagnoses. Finally, because of the increase in RBP, costs of RBP increase by 11%. Overall, in the period of five years, the introduction of this tool for BD screening could reduce the total and per-person costs by 3% of their initial value.

*Sensitivity analyses* To examine the model’s sensitivity to changes in model assumptions, a one-way sensitivity analysis was performed on selected variables (Table 2). Firstly, we varied the screening test’s sensitivity and specificity, where the low and high values corresponded to algorithm’s upper and lower 95% confidence intervals. The combination of low sensitivity/high specificity resulted in savings of $1681 per patient, while with high sensitivity/low specificity, we calculated savings of $822. After inputting low and high estimates of BD prevalence in patients newly diagnosed with MDD, we found that screening would save $518 and $2327 per patient, respectively. Next, we varied the annual probability of obtaining a correct BD diagnosis without screening and the probability of psychiatric referral upon positive a screening result. Annual diagnosis correction rates of 10% and 20% would save $1644 and $1228, respectively. Psychiatric evaluation in 50% cases would yield savings of $207, while 100% evaluation rate would result in $2638 savings per patient. Finally, we explored the effects of differences in direct costs for UBP as compared RBP and MDD, as well as savings if costs for RBP were equal to those of MDD patients. This was done by shifting both the values for relevant categories to 50% or 150%. High and low estimates of relative cost differences between RBP and UBP yielded savings of $1554 and $1291 per patient, respectively. When applied to high and low estimates of cost difference between MDD and UBP, this reflected in savings of $4985 if the difference was high, and an additional cost of $2139 if the difference was low. Assuming that the costs for RBP are equal to the costs of correctly diagnosed MDD, the model estimated per-person savings of $1864.

**Table 2:** One-way sensitivity analysis of discounted per-patient costs

Sensitivity analysis of discounted costs for patient presenting with new episode of MDD ($*)				
Original input parameters	Sensitivity analysis	No screening	Delta Study	Savings
Prevalence of UBP in new MDD cases (16%)				
	Low estimate (11%)	49,140	48,622	518
	High estimate (21%)	52,731	50,404	2327
Sensitivity & Specificity (0.76 , 0.87)				
	Low sensitivity, high specificity (0.58, 0.97)	50,936	49,255	1681
	High sensitivity, low specificity (0.92, 0.75)	50,936	50,114	822
Cost of RBP relative to UBP (− 7604)				
	High estimate (150%, − 11,406)	57,424	55,869	1554
	Low estimate (50%, − 3802)	44,448	43,157	1291
Cost of MDD relative to UBP (− 1936)				
	High estimate (150%, − 17,904)	74,718	69,732	4985
	Low estimated (50%, − 5968)	27,154	29,293	− 2139
Cost of RBP relative to MDD (+4332)				
	RBP = MDD	64,919	63,055	1864
Annual probability of diagnosis correction (15%)				
	Low estimate (10%)	51,402	49,758	1644
	High estimate (20%)	50,525	49,297	1228
Probability of psychiatric evaluation (75%)				
	Low estimate (50%)	50,936	50,728	207
	High estimate (100%)	50,936	48,297	2638

RBP: Recognised bipolar disorder patients; MDD: Major depressive disorder; UBP: Unrecognised bipolar disorder patients; CI: Confidence interval

*Discounted costs expressed as 2021 USD

*Comparison with the MDQ* An analogous decision analysis framework was used to calculate the economic impact and accuracy of the MDQ, and to compare it to the performance of the Delta Study algorithm (Table 3). An MDQ cut-off point of $$\ge$$11, corresponding to sensitivity of 0.56 and specificity of 0.92, was both the most profitable and most accurate. Implementation of MDQ for BD screening with this cut-off resulted in total direct costs of $745.8 million, or $49.717 per person, over five years. This represented five-year savings of $18.3 million, or $1219 per person, compared to no-screening. However, compared to the Delta Study algorithm, the total direct costs over five years for the MDQ were higher by $3.1 million ($204 per patient). In terms of accuracy, after five years 9214 patients were diagnosed correctly and 367 were diagnosed incorrectly using the MDQ. This represented 313 (3.3%) more correct diagnoses relative to no-screening, and 107 (1.1%) fewer correct diagnoses compared to the Delta Study algorithm.

**Table 3:** Delta Study algorithm and MDQ performance comparison at respective optimal cut-off points

Tool	Cut-off	Optimisation	Sensitivity	Specificity	PPV	NPV	Total per-person costs	Misdiagnoses (Y5)
Delta study	0.46	Misdiagnoses	0.87	0.81	0.46	0.97	$ 49,513	260
Delta study	0.56	Costs	0.76	0.87	0.52	0.95	$ 49,462	282
MDQ	$$\ge$$11	Costs & misdiagnoses	0.56	0.92	0.58	0.91	$ 49,717	367

PPV: Positive predictive value; NPV: Negative predictive value; Y5: Year 5 of followup; MDQ: Mood disorder questionnaire

The cost distribution plot in Fig. 3 indicates improved asset distribution for both the MDQ and the Delta Study algorithm compared to no-screening. While both tools were more cost-effective than no-screening at recognising UBP, the Delta Study algorithm featured a better ability to discern BD patients than the MDQ, with lower per-patient costs of misdiagnosis ($3679 vs. $5187), and a higher proportion of costs for RBP ($9043 vs. $7044). In turn, the MDQ’s higher specificity score at the cut-off of 11 resulted in a higher proportion of costs assigned to correctly diagnosed MDD patients compared to the Delta Study algorithm ($37,487 vs. $36,791).

### Decision curve analysis

**Fig. 4:**
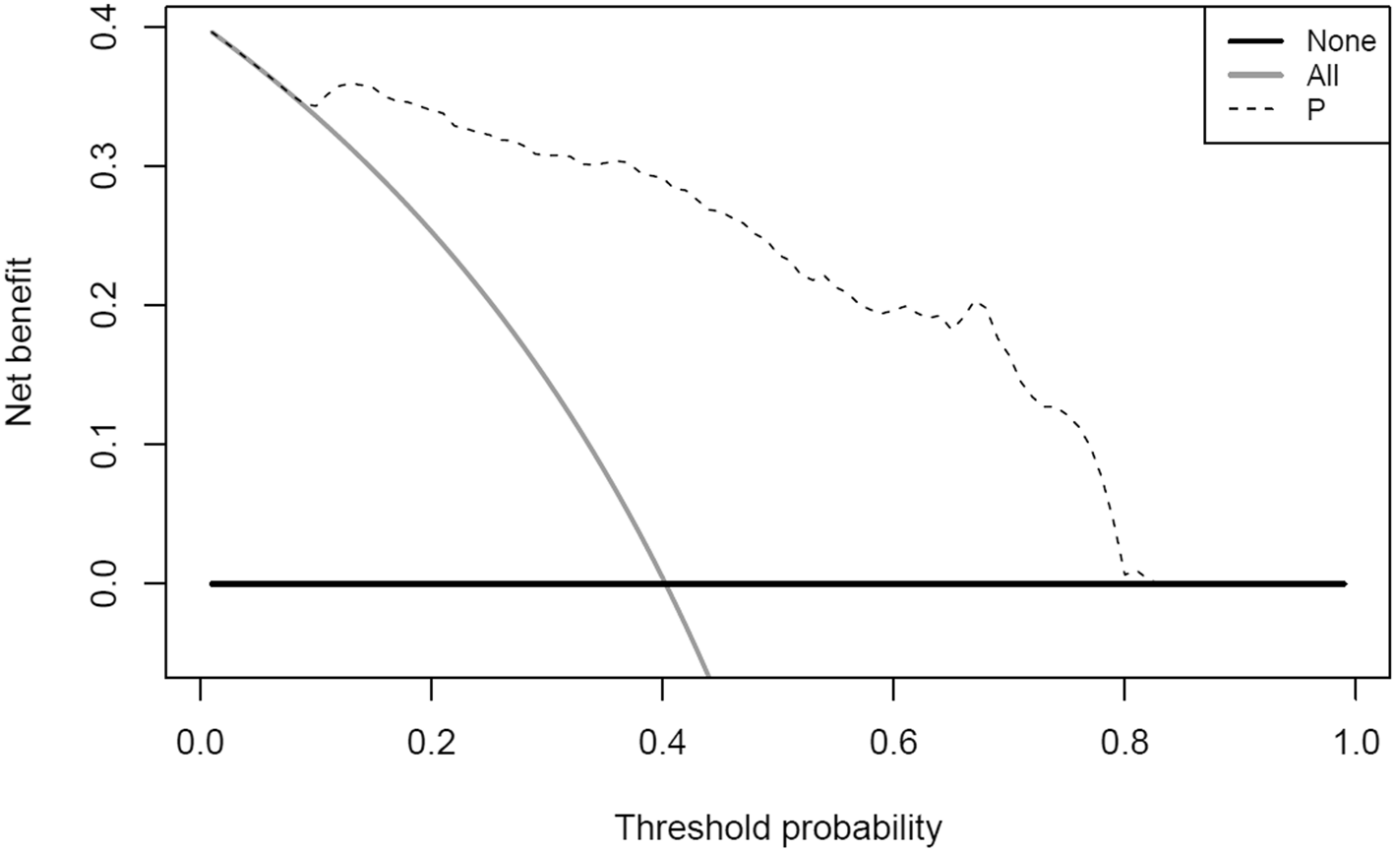
Decision curve analysis. Decision curve analysis plotting net benefit at all threshold probabilities for the Delta Study algorithm. Solid lines show the net benefit assuming that all patients are bipolar (grey), and assuming that all patients have MDD (black). The dotted line represents the net benefit of the Delta Study algorithm. MDD: Major depressive disorder

At both optimal cut-off points determined for the Delta Study algorithm, i.e. 0.46 and 0.56, the decision curve analysis indicated a higher net benefit compared to assuming that all patients are bipolar or that all have MDD (Fig. 4). Furthermore, the Delta Study algorithm shows an added utility not only at thresholds of interest, but also across the entire range of thresholds between 0.1 and 0.8.

## Discussion

The present study aimed to formulate the costs of misdiagnosis of BD as MDD, to evaluate the utility of using a newly developed diagnostic platform for screening for BD in patients who have been newly diagnosed with MDD, and to directly compare its performance with an established BD screening tool, the MDQ. We identified optimal diagnostic cut-offs in terms of the number of averted misdiagnoses and the associated cost-saving potential, and then evaluated the performance over a five-year period. Savings were achieved by averting an additional 4.4% of misdiagnoses, which is reflected in triaging of the otherwise misdiagnosed patients to less costly and more effective treatment journeys, appropriate for the patient’s true diagnosis. Overall, screening with the Delta Study algorithm resulted in reducing the expenditures by approximately 3% of the total costs, as well as achieving substantially fewer unrecognised cases. When compared to MDQ screening, the Delta Study algorithm achieved better results in all aspects, except for the number of correctly diagnosed MDD patients.

The decision analysis model used as a framework here was adapted from (Menzin et al. 2009), updating most of the assumptions with more recently obtained data. The updated model assumed a one-year incidence of MDD of 3%, which is a widely accepted estimate (Ferrari et al. 2013). Additionally, it was assumed that half of the patients who suffer from a depressive episode would seek medical help. This might have been a conservative estimate, considering that recent data show help-seeking rates in symptomatic individuals of 65.1% (Tomczyk et al. 2018). The prevalence of BD misdiagnosis in recent onset MDD patients was assumed to be 16%. This estimate was based on a large-scale published study using data from community and primary care settings (Angst et al. 2011). During the literature review, we found evidence indicating rates of BD misdiagnosis, ranging between 2% and 27.9% (Akiskal et al. 1995; Hughes et al. 2016), with a mean of 12.7%. That being said, the selected value comes from a multi-national and cross-cultural study featuring a large sample and utilising homogeneous methodology, and thus it is likely more representative than averaged values from multiple methodologically diverse studies. Similarly, although the attrition rate used for modelling was based on a median value from a 2016 report on attrition from healthcare plans (Government Accountability Office 2017), the values in the same report ranged between 1% and 39%, making this assumption valid for general modelling, but strongly context-dependent. Finally, the model assumed an average delay in diagnosis of six years, which is in accordance with previous reports showing values ranging between 5.4 and 7.5 years on average (Morselli and Elgie 2003; Ghaemi et al. 1999; Martin-Key et al. 2021).

The best performing cut-off in terms of reduction of misdiagnoses by the Delta Study algorithm featured superior precision in spotting unrecognised BD patients, but in turn identified fewer correctly diagnosed MDD patients than with no-screening or when screening with the MDQ. Although the Delta Study algorithm’s sensitivity of 87% is close to 90%, which is a desirable value for a screening tool (Zimmerman et al. 2011), using an algorithm with specificity lower than 100% will inevitably result in higher rates of false positive findings when compared to no screening. This is even despite the heavy emphasis on referral to a specialist for diagnostic confirmation upon receiving a positive result. Although the misdiagnosis of MDD could also have a negative impact, inappropriate treatment with mood stabilising medication is less likely to impact as negatively on an MDD patients’ mental health than in the case of potential antidepressant-induced mania. Furthermore, patients misdiagnosed with depression by the Delta Study algorithm are likely to present with substantial mood instability (possibly in the context of a bipolar spectrum disorder) and may in fact benefit from mood stabilising medication.

Despite the growing evidence that the introduction of digital screening tools into general medical practice would be highly beneficial (Hughes et al. 2016), its adoption into healthcare faces many challenges. Even despite the superior performance, administering the Delta screening tool in its current format is slightly more time consuming than already established shorter alternatives such as MDQ. Additionally, while innovative solutions such as Delta Study’s machine learning algorithm show good diagnostic accuracy in specific target groups used to develop the models, their performance in the general population is uncertain and requires thorough evaluation (Shatte et al. 2019). Certainly, the application of screening tools in mental healthcare requires careful validation and oversight, with its greatest potential lying in using it in conjunction with more established methods as part of the clinical triage process, or before prescribing medication to harder to diagnose cases. Such an integrated approach would facilitate earlier, more accurate, standardised and cost-effective diagnoses of diverse mental health conditions. As supported by the present analyses, the adoption of screening tool into mental healthcare could not only reduce the number of misdiagnoses, but also reflect positively in terms of economic impact, saving millions of dollars.

The findings from the present study should be interpreted within their limitations in mind. The Delta Study’s online recruitment strategy of participants may limit generalisability to other clinical settings. Although the algorithm has been extensively validated with good results, it would need to be trialed in the intended clinical settings for a full evaluation of its validity. Additional limitations stem from the study’s exclusive use of secondary sources to estimate direct costs, particularly in the view of the surprisingly limited published literature on the subject. Thus, a significant limitation could be the potentially outdated assumption of the costs of misdiagnosis. Since the time the costs were estimated, there have been substantial advances in mental healthcare. This includes various new medications for the treatment of BD (Rhee et al. 2020), but also the revision of healthcare guidelines (Bessonova et al. 2020), as well as the introduction of two federal laws (Mental Health Parity and Addictions Equity Act and Affordable Care Act) (Frank et al. 2014), making mental healthcare more affordable and accessible. As such, the exact costs as reported here might not reflect the current costs and their distribution across the diagnostic groups, despite an adjustment for inflation. Furthermore, our analyses were focused on lowering the rates of misdiagnosis, but the aspect of reducing the deterioration of a given patient’s mental health was not taken into account. As such, the costs remained unchanged over time, whereas some degree of cost reduction can be expected due to a stabilised mental state of treated patients. Finally, due to a lack of information about the costs of misdiagnosis of MDD as BD, we assumed the cost to be equal to that of misdiagnosis of BD as MDD. However, in reality, one would expect less severe consequences of misdiagnosis of MDD, due to mood stabilisers not worsening the condition to such a significant degree as would antidepressant monotherapy in BD. Taken together all our assumptions are conservative and may substantially underestimate the potential clinical and financial impact.

## Conclusions

In conclusion, the present study highlights the potential impact of one-off screening for BD in patients presenting with recent onset depressive symptoms in the primary care setting. The incorporation of novel screening tools with good performance to identify misdiagnosed BD patients shows promising results when compared to either the no-screening scenario or screening using the MDQ. We suggest that screening for BD and could lead to a significant reduction in the number of misdiagnosed patients, reduce direct healthcare costs, and most importantly facilitate earlier diagnosis, reduce suffering, and promote better outcomes.

## Supplementary Information

**Additional file 1.** **Figure S1.** Delta Study algorithm's performance as expressed by sensitivities and Specificities (*y* axis), as well as their respective 95% confidence intervals for all the cut-off points (*x* axis). **Figure S2.** MDQ performane as expressed by sensitivities and specificities (*y* axis), as well as their respective 95% confidence intervals for all the cutoff points (*x* axis). **Figure S3.** Schematic depiction of the decision analysis model based on Menzin et al. (2009). Ovals represent a chance node, diamonds represent an outcome with a chance of changing, and sharp- and soft-edged rectangle nodes represent start and end states, respectively. Key: BD = Bipolar disorder. MDD = Major depressive disorder. UBP = Unrecognised bipolar disorder patients. RBP = Recognised bipolar disorder patients.

## Data Availability

All data generated or analysed during this study are included in this published article [and its supplementary information files]

## Abbreviations

BD: Bipolar disorder
MDD: Major depressive disorder
SSRI: Selective serotonin reuptake inhibitor
DSM-5: Diagnostic and Statistical Manual of Mental Disorders, 5th Edition
ICD-11: International Statistical Classification of Diseases and Related Health Problems, 11th Revision
NICE: National Institute for Health and Care Excellence
MDQ: Mood disorder questionnaire
CIDI: Composite International Diagnostic Interview
ROC-AUC: Receiver operating characteristic curve
RBP: Recognised bipolar patients
UBP: Unrecognised bipolar patients
PPV: Positive predictive value
NPV: Negative predictive value
