# Supplementary material for: Using decision-analysis modelling to estimate the economic impact of the identification of unrecognised bipolar disorder in primary care: the untapped potential of screening
Source: Int J Bipolar Disord. 2022 Jun 10;10:15. doi: 10.1186/s40345-022-00261-9 (PMC9184689; doi:10.1186/s40345-022-00261-9)
Supplement: Supplementary file 1 — Additional file 1. Figure S1. Delta Study algorithm's performance as expressed by sensitivities and Specificities (y axis), as well as their respective 95% confidence intervals for all the cut-off points (x axis). Figure S2. MDQ performane as expressed by sensitivities and specificities (y axis), as well as their respective 95% confidence intervals for all the cutoff points (x axis). Figure S3. Schematic depiction of the decision analysis model based on Menzin et al. (2009). Ovals represent a chance node, diamonds represent an outcome with a chance of changing, and sharp- and soft-edged rectangle nodes represent start and end states, respectively. Key: BD = Bipolar disorder. MDD = Major depressive disorder. UBP = Unrecognised bipolar disorder patients. RBP = Recognised bipolar disorder patients. [file 40345_2022_261_MOESM1_ESM.pdf]

Supplementary materials

A. Screening method performance

A.1. Delta study algorithm performance

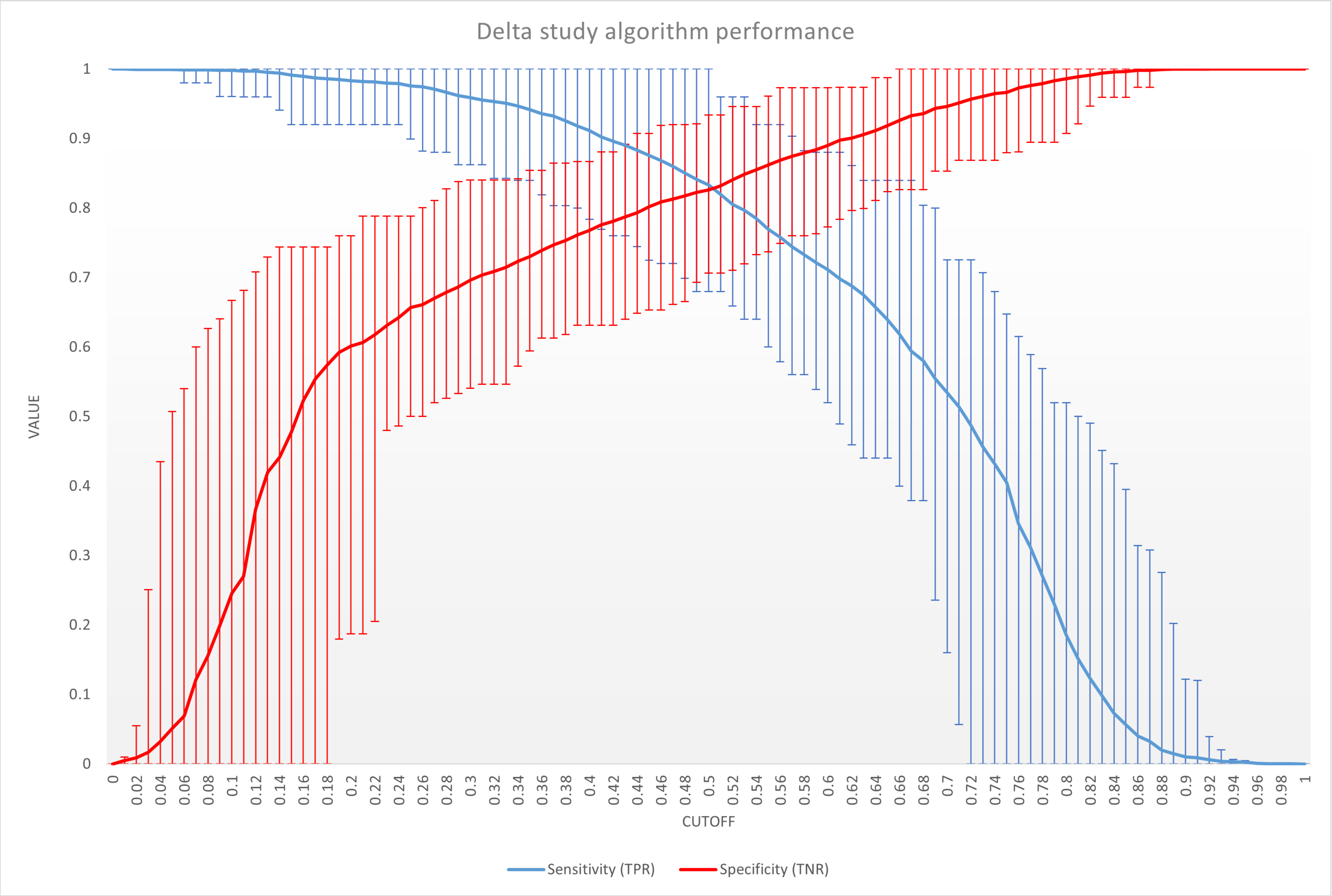

Figure S1. Delta Study algorithm's performance as expressed by sensitivities and Specificities (y axis), as well as their respective 95% confidence intervals for all the cut-off points (x axis)

A.2. MDQ performance

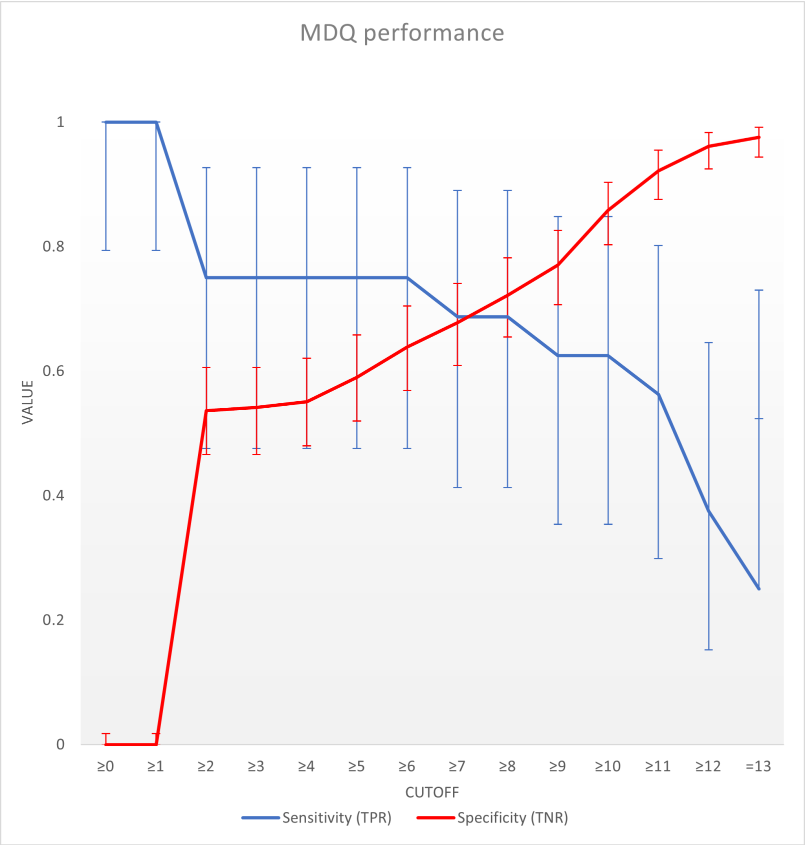

Figure S2. MDQ performance as expressed by sensitivities and specificities (y axis), as well as their respective 95% confidence intervals for all the cutoff points (x axis)

B. Model Structure

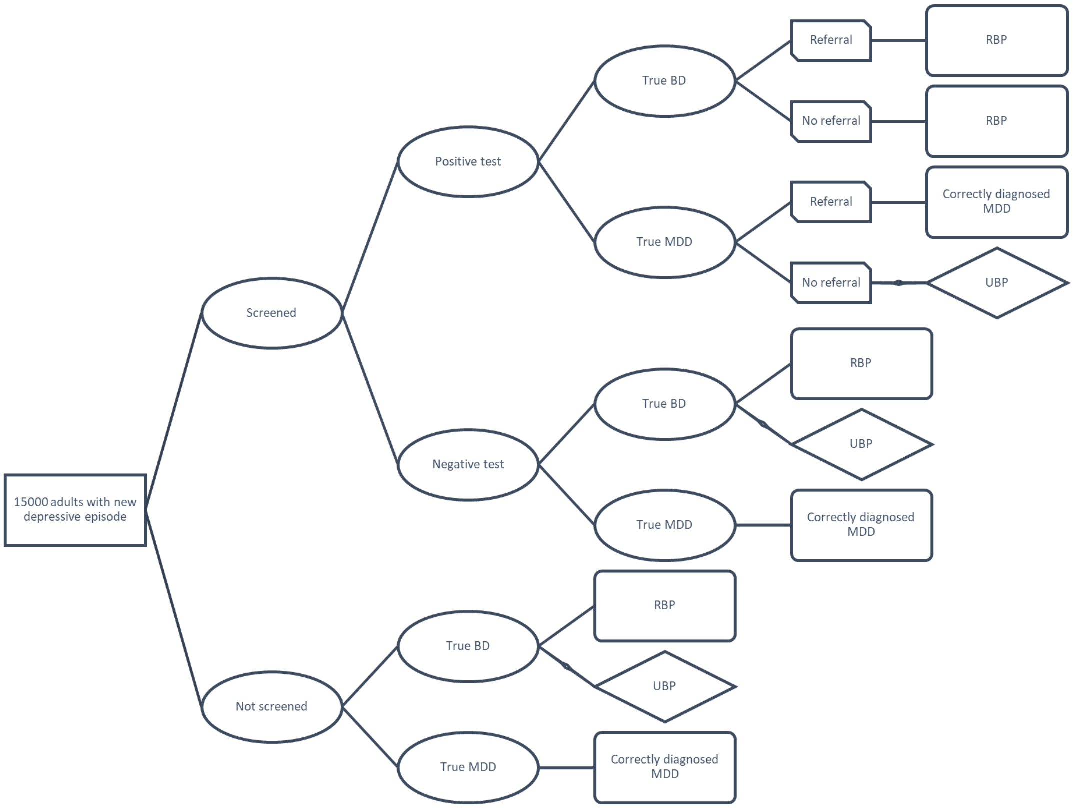

Figure S3. Schematic depiction of the decision analysis model based on Menzin et al. (2009). Ovals represent a chance node, diamonds represent an outcome with a chance of changing, and sharp- and soft-edged rectangle nodes represent start and end states, respectively.
Key: *BD = Bipolar disorder. MDD = Major depressive disorder. UBP = Unrecognised bipolar disorder patients. RBP = Recognised bipolar disorder patients*

References

[1] Menzin, J., Sussman, M., Tafesse, E., Duczakowski, C., Neumann, P., & Friedman, M. (2009). A model of the economic impact of a bipolar disorder screening program in primary care. *The Journal of clinical psychiatry*, 70(9), 0–0.
